# Neurological Complications Following Temporomandibular Joint Injections in Patients with Temporomandibular Disorders: A Systematic Review of Reported Adverse Events

**DOI:** 10.3390/jcm14165770

**Published:** 2025-08-15

**Authors:** Maciej Chęciński, Kamila Chęcińska, Izabella Chyży, Kamila Walkowiak, Natalia Turosz, Bartosz Kosiński, Sebastian Zduński, Dariusz Chlubek, Maciej Sikora

**Affiliations:** 1 National Medical Institute of the Ministry of the Interior and Administration, Wołoska 137 Str., 02-507 Warsaw, Poland; maciej.checinski@pimmswia.gov.pl (M.C.); kamila.checinska@pimmswia.gov.pl (K.C.); natalia.turosz@gmail.com (N.T.); sebastian.zdunski@pimmswia.gov.pl (S.Z.); sikora-maciej@wp.pl (M.S.); 2 Department of Maxillofacial Surgery, Hospital of the Ministry of the Interior and Administration, Wojska Polskiego 51, 25-375 Kielce, Poland; 3 Department of Anesthesiology and Intensive Care, Leszek Giec Upper-Silesian Medical Center, Medical University of Silesia in Katowice, Ziołowa 45–47, 40-635 Katowice, Poland; ichyzy@gcm.pl (I.C.); kwalkowiak@gcm.pl (K.W.); 4 Faculty of Architecture, Civil Engineering and Applied Arts, Academy of Silesia, Rolna 43, 40-555 Katowice, Poland; bartosz.kosinski@akademiaslaska.pl; 5 Department of Biochemistry and Medical Chemistry, Pomeranian Medical University, Powstańców Wielkopolskich 72, 70-111 Szczecin, Poland

**Keywords:** temporomandibular joint, intra-articular injections, adverse effects, facial nerve diseases, arthrocentesis

## Abstract

**Background:** Temporomandibular joint (TMJ) injections and arthrocentesis are commonly used minimally invasive methods for treating temporomandibular disorders (TMDs). Although considered safe, they can cause neurological complications. The aim of this systematic review was to synthesize all identified evidence for neurological adverse events following intra-articular TMJ interventions. **Methods:** This review was based on a systematic search with BASE, DOAJ, PubMed, SciELO, and Semantic Scholar on 28 May 2025. It included primary studies involving patients diagnosed with TMDs who underwent intra-articular injections into the TMJ or were treated with arthrocentesis, and in whom neurological adverse effects associated with the intra-articular intervention were reported. Studies reporting non-specific symptoms or unrelated systemic conditions were excluded. The risk of bias was assessed using the Joanna Briggs Institute’s critical appraisal tools. Results were presented in summary tables. **Results:** The search yielded five eligible studies comprising 319 patients, of whom 320 neurological adverse events were reported. Included studies comprised a randomized controlled trial, two retrospective studies, and two case reports. Four studies had a low risk of bias, and one had a moderate risk of bias according to the Joanna Briggs Institute appraisal tools. The proportion of patients affected ranged from 14% to 65% depending on the study design and intervention type. The most common adverse event was transient facial nerve (cranial nerve VII) paralysis, mainly involving the temporal and zygomatic branches. Less commonly reported complications involved the trigeminal nerve branches (V1, V3). There is also a single case of epidural hematoma with palsy of the oculomotor nerve (III). Most symptoms resolved spontaneously within a few hours to a few days. The use of local anesthesia and large volumes of irrigation (60 mL) during arthrocentesis increases the risk of complications. Attempts to explain the mechanisms of complications include local anesthetic diffusion, compression neuropraxia due to lavage fluid leakage, and corticosteroid neurotoxicity. One of the limitations of the study is the scarcity of data. **Conclusions:** Although most adverse events are mild and reversible, these findings highlight that precise, real-time guided injection and careful control of lavage volumes can minimize extra-articular spread of anesthetics or fluids, thereby reducing the likelihood of neurological complications. This study received no funding. PROSPERO ID number: CRD420251088170.

## 1. Introduction

The temporomandibular joint (TMJ) enables mandibular movement relative to the temporal bones and is essential for mastication, speech, and swallowing. Temporomandibular joint disorders (TMDs) are a collective name for various deviations from the physiological norm in the functioning of the TMJ. TMDs manifest themselves with acoustic symptoms, pain, and limited mobility. In more advanced cases, there is degradation of both the structure of the articular disc and the articular surfaces on the mandibular head and temporal bone [1,2,3,4,5].

Treatment approaches range from conservative therapy to invasive interventions such as intra-articular injections, joint cavity rinsing, arthroscopic surgery, or even joint replacement with a prosthesis [2,5,6,7,8,9,10,11].

Intra-articular injections are an increasingly popular method of TMD treatment. Due to their invasiveness, they are an alternative to conservative treatment, especially in cases where it has ceased to be effective. Intra-articular injections are used to reduce inflammation and, consequently, relieve pain and improve joint mobility. Hyaluronic acid and blood products administered as autografts are primarily used for this purpose. Different substances reduce the symptoms of TMD in different ways. For example, an important mechanism of action of hyaluronic acid is the lubrication of joint surfaces, autologous products (e.g., platelet-rich plasma, injectable platelet-rich fibrin) have a chondroprotective potential, and inflammation reduction may be achieved by administering drugs [1,2,5,6,12,13].

Although injections into the TMJs are generally considered safe, they can lead to rare but potentially serious complications, including neurological complications. Neurological complications include symptoms such as paralysis, paresis, hypoesthesia, or paresthesia in the area of innervation. The mechanisms of these complications may include damage to the nerve by the needle, toxic effects of the administered substances, or secondary edema leading to compression of the nervous structures. These mechanisms were proposed by the authors of the included studies and represent hypotheses rather than definitive causal determinations. The facial nerve, the auriculotemporal nerve, and the anterior branch of the great auricular nerve run in the area of TMJ injection, and each of them can potentially become the subject of one of the complications discussed. Although these cases are relatively rare in the context of TMJ injections, their potential burden and impact on patients’ quality of life deserve further investigation [14,15,16,17,18,19].

The anatomical proximity between the TMJ, parotid gland, orbit, and cranial cavity poses a potential risk for a variety of complications. Physiological and nonphysiological connections between anatomical spaces facilitate the spread of anesthetics or irrigating fluid. This increases the risk not only of unintended neural dysfunction but also of mechanical and chemical effects of the injected agent on other structures, such as connective tissue. Excessive intracranial or intraorbital pressure is a condition that requires immediate intervention and, while unlikely as a consequence of TMJ injection therapy, is potentially very serious [20,21,22,23].

Despite the increasing use of intra-TMJ injections, neurological complications have not been systematically summarized. Existing publications are limited to case reports or series, which do not allow for a broader analysis of mechanisms or preventive measures. This review fills this gap by systematically identifying, for the first time, all available evidence regarding neurological complications of intra-articular temporomandibular joint surgery, analyzing the mechanisms, and proposing preventive measures.

This review is intended to synthesize the available scientific literature on neurological complications resulting from intra-articular injections involved in the TMD treatment. The analysis focuses on identifying the complication types and severity, exploring potential risk factors, and discussing the mechanisms of their occurrence. These results may improve the safety of clinical practice and provide a better understanding of this rare but clinically significant group of complications.

Null hypothesis: The volume of the intra-articularly administered agent has no effect on the occurrence of neurological complications.

## 2. Materials and Methods

This study was conducted following the Preferred Reporting Items for Systematic Reviews and Meta-Analyses guidelines. It was registered in the International Prospective Register of Systematic Reviews (PROSPERO) under the number: CRD420251088170 [24,25].

The review was conducted to systematically compile and assess available evidence on neurological complications following intra-articular TMJ procedures, aiming to inform clinical decision-making and preventive strategies.

### 2.1. Eligibility Criteria

Studies involving patients diagnosed with TMDs were included in the review. Papers describing treatment with TMJ lavage or intra-articular injections into the TMJ or the TMJ area (e.g., in the bilamellar zone) were included. Studies involving more invasive concurrent procedures (e.g., arthroscopy, open surgery) were excluded.

For inclusion in this review, no comparator was required in a given study; however, a comparative meta-analysis of the incidence and severity of neurological complications between the following groups was planned as follows: (1) placebo injection (e.g., saline or Ringer’s solution), (2) injection of an active substance (e.g., hyaluronic acid, platelet-rich plasma, platelet-rich fibrin injection, steroid, hypertonic dextrose, etc.), (3) joint lavage (arthrocentesis). A comparative analysis was initially planned, but could not be performed because of insufficient homogeneous data across the included studies.

The specific inclusion criterion for this review was the occurrence of at least one neurological symptom as a consequence of injection therapy in the presented group of patients. Neurological complications were defined as any nerve-related adverse events reported in the included studies, ranging from transient sensory disturbances (e.g., paresthesia, numbness) to persistent motor deficits (e.g., facial nerve palsy). Both minor and transient symptoms and more severe manifestations were considered eligible, provided they were explicitly described as neurological in origin by the study authors. The following were taken into account: (1) consequences of anesthesia for injection therapy, (2) mechanical action of the needle or fluid during intra-articular injection, and (3) the effect induced by the introduced substance. Pain as a sole symptom without accompanying sensory or motor deficit (unless the investigators made a diagnosis of neuralgia) and symptoms caused by a known comorbidity (e.g., diabetes, shingles) were excluded.

There were no restrictions on the type of study design, thus allowing studies with varying degrees of evidence, including (1) randomized controlled trials, (2) non-randomized controlled trials, (3) uncontrolled studies and case series, (4) single case reports. The formal summary of inclusion and exclusion criteria for selection purposes is presented in Table 1.

### 2.2. Information Sources and Search Strategy

The identification of complications related to intra-articular TMJ injections and the development of search terms were described in our previous publication. The present systematic review used the keywords established in that work [23].

Initial searches were conducted using PubMed and Bielefeld Academic Search Engine (BASE). For transparency and the ability of readers to repeat searches, it was decided to use only open-access engines. Finally, the following electronic databases and platforms were searched: (1) Bielefeld Academic Search Engine (BASE), (2) Directory of Open Access Journals (DOAJ), (3) National Library of Medicine (PubMed), (4) Scientific Electronic Library Online (SciELO), and (5) Semantic Scholar. No filters or other modifiers were available as tools of the individual search engines were applied. All final searches were conducted on 28 May 2025.

Based on the above-mentioned eligibility criteria, subsequent queries were developed, and after completing the initial searches, the final search strategy was formulated as follows:

“(temporomandibular OR tmj OR tmd) AND (injection OR injections OR intra-articular OR intraarticular OR intra-cavitary OR intracavitary OR arthrocentesis OR rinse OR rinsing OR lavage) AND (paralysis OR paresis OR hypoesthesia OR paresthesia OR dysesthesia OR neuropathy)”.

### 2.3. Selection and Data Processing

The authors (M.C., K.C.) performed blinded selection against the eligibility criteria based on the titles and abstracts. In case of discrepancies, the given record was promoted to full-text review on an equal footing with the unanimously approved ones. The full-text evaluation was performed on the same basis. No automation tools were used for selection. The same authors conducted a fully manual extraction into tables.

The following data were collected: (1) data identifying a given paper (first author, year of publication, unique identifier), (2) number of patients with neurological complications and total number of patients, (3) diagnosis (e.g., internal derangement, osteoarthrosis, degenerative joint disease, hypermobility), (4) type of intervention (arthrocentesis, injection, or combination), (5) injected substance (e.g., saline, Ringer’s solution, hyaluronic acid, platelet-rich plasma, hypertonic dextrose), (6) number of injections, (7) volume per injection, (8) total volume administered, (9) type of complication, (10) information on resolution of the complication (transient with duration or persistent during the follow-up period with an indication of the length of follow-up), and (11) recurrence (in the case of resolution during the follow-up period).

The extracted data were organized into tables. A tabulated meta-synthesis was then conducted, arranged by cranial nerve affected. The synthesis included the number of previously reported cases of each complication and presented the proposed mechanism of action, including the interpretations provided by the authors of the individual source articles.

### 2.4. Study Risk of Bias Assessment

The risk of bias was assessed using the Joanna Briggs Institute’s critical appraisal tools, which are specifically tailored to address various types of research, including randomized controlled trials, cross-sectional studies, and case reports [26]. These standardized checklists facilitate a critical evaluation of research studies by analyzing key methodological aspects.

The assessment was performed independently by two reviewers (M.C., I.C.). The inter-rater agreement was calculated using Cohen’s kappa statistic. All discrepant assessments were resolved through discussion until a consensus was reached.

## 3. Results

### 3.1. Study Selection

**Figure 1:**
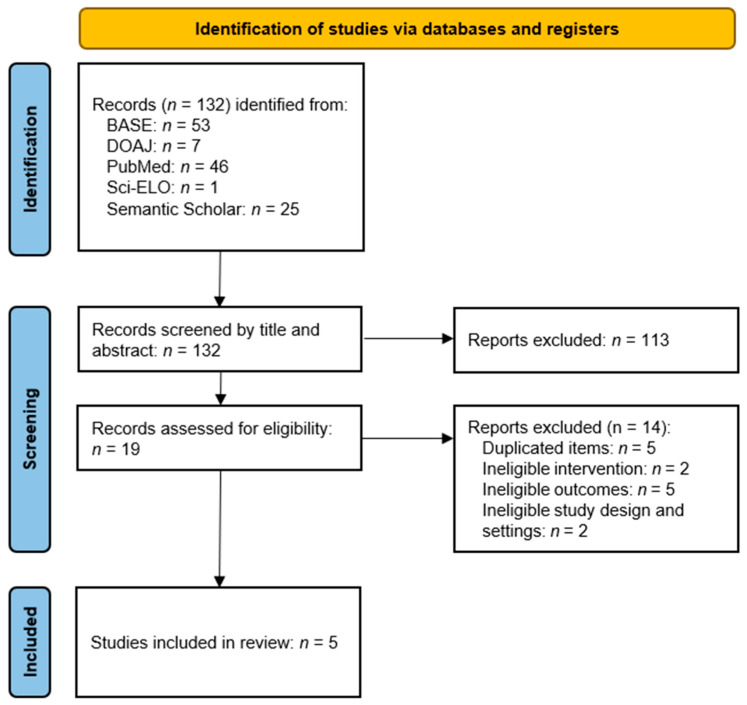
Flow diagram.

The selection process is presented in Figure 1. Of the 132 records identified (Table A1), 113 were excluded based on the content of their titles and abstracts. The remaining 19 were deduplicated, where five records were rejected as duplicates. The resulting set was evaluated in full-text form, leading to further exclusions (Table A2).

### 3.2. Study Characteristics

This systematic review included five studies published between 2000 and 2025. Two retrospective studies, two case reports, and a randomized controlled trial were incorporated. Their brief characteristics are presented in Table A3 [27,28,29,30,31].

### 3.3. Risk of Bias in Studies

The assessment of bias risk revealed that four studies had a low risk of bias [27,28,29,30]. The studies conducted by Baş et al. and Aliyev et al. fulfilled all the criteria of the Joanna Briggs Institute critical appraisal checklist, indicating high methodological quality [27,29]. Only one study demonstrated a moderate risk of bias due to the lack of identification and management of confounding factors and unclear statistical analysis [31]. The inter-rater agreement was almost perfect (κ = 0.82), indicating very good consistency between reviewers. A detailed assessment is presented in Table A4, Table A5 and Table A6.

### 3.4. Results of Individual Studies

Table 2 summarizes studies that reported neurological complications after intra-articular temporomandibular joint procedures. Both drug injections and joint irrigation with solutions, often combined with auriculotemporal nerve blockade, were included. Details of the intervention and complications observed are provided for each study [27,28,29,30,31].

Neurological complications can involve both sensory (e.g., ophthalmic or mandibular nerve) and motor (e.g., facial nerve) nerves and are usually transient. The most serious complication reported was an epidural hematoma with oculomotor nerve palsy, which also resulted in full recovery after neurosurgical treatment. In most cases, symptoms resolved spontaneously within hours or days, suggesting a dominant effect of the local anesthetic as the main mechanism of these complications [27,28,29,30,31].

### 3.5. Results of Syntheses

Owing to substantial heterogeneity in study designs, intervention protocols, and outcome definitions, a formal meta-analysis was not feasible, and results are presented descriptively.

Table 3 summarizes neurological complications identified in the analyzed material, grouped by damaged cranial nerves. For each nerve, the number of recorded cases and the presumed mechanism of the disorders are given [27,28,29,30,31].

The analysis indicates that most of the described complications came from Baş et al. and Vaira et al. and concerned the facial nerve (VII), where the effects of the local anesthetic led to a transient blockade of motor functions. Complications related to the ophthalmic (V1) and mandibular (V3) branches of the trigeminal nerve were also reported, most likely resulting from the anesthetic effect. An exceptional complication, described only once, was an epidural hematoma, which manifested as contralateral hemiparesis and palsy of the unilateral oculomotor nerve [27,28,29,30,31].

## 4. Discussion

### 4.1. General Interpretation

This review found that most neurological complications following intra-articular TMJ procedures are transient facial nerve palsies, with a single report of epidural hematoma. The small number of studies and predominance of non-randomized designs limit the strength of conclusions.

The identified data on adverse neurological events associated with minimally invasive manipulations of the TMJ are scarce. It may be suspected that they are reluctantly reported or their reporting is limited to a brief mention in the full text of the article, which limits indexing and does not allow for identification through literature searches. In the included retrospective and controlled studies, the percentage of adverse events of the nature of excessive anesthesia is significant and amounts to up to 65%. Therefore, it should be suspected that this may be more frequent than currently reported, but robust data are lacking. However, due to its transience with the wear off of the anesthesia effect and its low inconvenience, it may be omitted in the reports of many investigators [27,28,29,30,31].

All reported complications were related to the TMJ irrigation procedure or intra-articular corticosteroid injection. The most commonly affected nerve is the facial nerve (cranial nerve VII), especially its temporal and zygomatic branches, probably due to the diffusion of local anesthetic and involvement of neural branches that were not intended to be anesthetized. Another possible mechanism is neuropraxia from compression resulting from extravasation of irrigating fluids outside the joint cavity [27,29,30,31].

While most complications are minor and self-limiting, one serious complication has been reported—an epidural hematoma, which is an exceptional case but provides an idea of the risks associated with the proximity of the cranial cavity. The nature of this complication was not completely explained. Lactated Ringer solution 60–80 mL was used at that time, which could presumably be a volume sufficient to penetrate the cranial cavity (provided that an appropriate connection is created) and mechanically cause separation of the dura mater from the inner surface of the temporal bone. In such a scenario, it is natural that the resulting cavity, primarily filled with lactated Ringer solution, is replaced with blood over time. Another considered mechanism of this complication is laceration of a vessel large enough that the resulting bleeding was sufficient to produce the described hematoma. Nevertheless, the hypotheses of mechanisms remain speculative and not supported by direct anatomical or clinical evidence [28,32].

Apart from the above-mentioned single case of epidural hematoma, in the course of this review, no other neurological complications of TMJ injections or irrigations more serious than transient nerve conduction disturbance were identified in the world literature. The latter particularly concerned the temporal and zygomatic branches of the facial nerve, following their anatomical proximity. Apart from the hematoma, where the method of patient anesthesia was not presented, in all cases of neurological complications, intra-articular intervention was preceded by local anesthesia, often specified as auriculotemporal nerve block [27,29,30,31].

The use of an anesthetic before TMJ injection is not obvious. Some research teams administer substances intra-articularly without prior anesthesia of the area undergoing the procedure. This approach eliminates the risk of sensory disturbances and temporary motor block resulting from the diffusion of the anesthetic. The situation is different with double-needle (and presumably also double-lumen) arthrocentesis, where the amount of fluid passed through the joint cavity strongly supports the use of anesthesia. Furthermore, Baş et al. assume that not only the anesthesia itself, but also the leakage of the rinsing fluid outside the joint cavity may be the cause of neurological disorders, which they explain by mechanical compression and neuropraxia [6,12,27,31,33,34].

### 4.2. Future Perspectives

Neurological complications of intra-articular injections are primarily related to (1) the use of local anesthesia, (2) rinsing the joint cavity with at least 60 mL using the double-needle method, or (3) the administration of corticosteroids. Although they are transient, they may suggest irregularities in the technique itself. Excessive local anesthesia, involving subsequent nerve branches, is not the only possible mechanism of neurological complications typical of minimally invasive manipulations associated with TMJ. Leakage of rinsing fluid outside the joint cavity, resulting in neuropraxia and corticosteroid neurotoxicity, is another realistically considered mechanism of action leading to complications. Its occurrence suggests an imperfection of the blind injection technique into the joint cavity and even the presumably common but unaware extra-articular administration. Alternative etiologies should be considered in the differential diagnosis, including surgical, infectious, and inflammatory processes affecting the head and neck region [27,29,30,31,35].

In the context of the above considerations, the following strategies can be considered to reduce the risk of extra-articular deposition or its sequelae:

(1) Minimize the risk of complications by administering a smaller volume—choose injection rather than arthrocentesis if possible.
(2) Choose substances safer with regard to deposition in an unintended location, including extra-articular or even intravascular.
(3) Attempt real-time guided deposition or per surgical template, although these are theoretical solutions, as the ultrasonography so far tested in this indication does not give unequivocal results [36,37,38].

Therefore, clinicians performing TMJ injections or arthrocentesis should consider using the minimal effective volume, employ meticulous technique to reduce extra-articular spread, and document any neurological symptoms. Future studies should use standardized complication definitions, report detailed procedural parameters, and evaluate preventive strategies such as image guidance.

### 4.3. Strengths

The systematic review was designed to ensure reliability, transparency, and reproducibility. Neurological complications associated with temporomandibular joint injections are rarely discussed in the literature, which makes this review innovative and potentially fills an important knowledge gap. The review has clinical significance, providing practical information for dentists, maxillofacial surgeons, and specialists treating TMJ disorders, helping to improve the safety of the procedures used. Another strength is the broad perspective that the review takes, including different injection techniques, such as intra-articular corticosteroids, hyaluronic acid, or platelet-rich plasma, as well as joint irrigation procedures.

### 4.4. Limitations

The data on neurological complications of TMJ injections and lavage are scarce. The incidence of neurological complications may be relatively high, but their reporting is unsatisfactory. There is a potential risk of publication bias, as studies reporting rare complications may be less likely to be published in the scientific literature. The heterogeneity of the methodologies used in the included studies, including differences in study designs, injection techniques, types of substances used, and diagnostic criteria for complications, makes it difficult to compare results and draw firm conclusions.

In addition to publication bias, potential sources of bias include language bias (eligibility required an English-language abstract), selection bias due to manual screening, reporting bias in case reports and small series, and the lack of long-term follow-up data. Due to the small number of studies and their heterogeneity, a formal assessment of publication bias (e.g., funnel plot asymmetry or Egger’s regression test) was not feasible.

Given the finite list of medical article databases searched with the selected engines, there is a risk that papers published in less well-indexed journals were missed. The search strategy was intentionally limited to open-access databases to ensure replicability of the review. However, this approach may have led to the omission of relevant studies indexed solely in restricted-access databases. Manual screening and extraction, without the use of automation tools, may introduce selection bias and reduce reproducibility. This approach was adopted intentionally, as AI-based automation tools for study selection are still considered more error-prone, whereas human screening remains the gold standard in systematic reviews.

## 5. Conclusions

(1) Neurological complications following intra-articular injections for TMD are rarely described but potentially common, especially when local anesthesia and/or joint irrigation with 60 mL or more of fluid using the double-needle technique are used.
(2) The most common are transient facial nerve palsies (temporal and zygomatic branches), resulting from the spread of the anesthetic agent or pressure from fluid leakage outside the joint cavity.
(3) These mechanisms may result from an imprecise technique, suggesting the need for careful deposition and developing navigation to optimize clinical safety.
(4) Although most complications are mild and self-limiting, one case of epidural hematoma has been reported.

The conclusions of this review are based on a very limited number of studies, most of which have methodological constraints. Therefore, the certainty of evidence regarding neurological complications following intra-articular TMJ interventions is very low, primarily due to a low number of source studies, their observational designs, and small sample sizes.

## Figures and Tables

**Table 1:** Summary table of study inclusion and exclusion criteria.

Framework Component	Criteria for Inclusion	Criteria for Exclusion
Patient population	Patient with TMDs	Cadaver or animal studies
Index intervention	TMJ intra-articular injection	More invasive concurrent procedure
Comparator (included in selected analyses only)	Placebo, active substance injection, or arthrocentesis	Not applicable
Outcomes	Neurological adverse effects related to intra-articular injections	Non-specific symptoms or unrelated systemic conditions
Study design and settings	Articles on primary studies	No English abstract available

**Table 2:** Summary of studies reporting neurological complications following intra-articular interventions for TMJ disorders.

First Author, Publication Year	Diagnosis per Authors	Substance and Intervention	Intra-Articular Injection Volume	Neuro Complications (n/N)	Type and Course of Complication	Duration (Until Resolution or End of Observation)
Baş, 2025 [27]	Painful intra-articular temporomandibular disorders	Lidocaine local anesthesia and lactated Ringer solution two-needle arthrocentesis	60–100 mL in one session	30/210	Transient facial nerve paralysis (mainly temporal and zygomatic branches)	Resolved within a few hours
Carroll, 2000 [28]	TMJ dysfunction	Lactated Ringer solution arthrocentesis	60–80 mL in one session	1/1	Right-sided extradural haematoma with third nerve palsy and left hemiparesis; required craniotomy and clot evacuation; full neurological recovery	Neurological symptoms resolved within 2 weeks after craniotomy; 3-month follow-up was uneventful
Aliyev, 2019 [29]	Severe bruxism causing painful jaw locking and trismus in the right TMJ	Intra-articular articaine injection and two-needle arthrocentesis with lactated Ringer solution	2.5 mL articaine hydrochloride, 102–202 mL lactated Ringer solution (administration stopped during the second 100 mL portion)	1/1	Transient right-sided facial nerve paralysis; anesthesia of the inferior alveolar and lingual nerves	Resolved within 24 h (symptoms observed for ~2 h post-op; no complications on next-day follow-up)
Isacsson, 2019 [30]	Unilateral TMJ arthralgia without sounds in the affected joint	Auriculotemporal nerve block with 1.8 mL prilocaine-felypressin and intra-articular methylprednisolone 40 mg/mL	1 mL in one session	3/27 cases of eyelid paresthesia and 2/27 cases of numbness (overlap between cases not reported)	Transient paresthesias and numbness	Resolved during 4-week follow-up (exact timing not specified)
Vaira, 2018 [31]	Temporomandibular disorders	Auriculotemporal nerve block with mepivacaine with adrenaline; two-needle arthrocentesis with lactated Ringer solution; intra-articular sodium hyaluronate	100 mL lactated Ringer solution and 1.5–2 mL sodium hyaluronate	282/433 procedures in 315 patients	Transient frontalis and orbicularis oculis paresis (facial nerve)	Fully resolved with the end of the local anaesthesia effect

n—number of patients with neurological complications; N—total number of patients in the study.

**Table 3:** Neurological complications per cranial nerve in the entire dataset.

Cranial Nerve	Number of Cases	Assumed Mechanism
III (Oculomotor)	1	(1) Extradural hematoma due to vascular injuryor or (2) Solution leakage
V1 (Ophthalmic branch of trigeminal)	3	(1) Local anesthetic effect causing sensory disturbances (2) Administration of corticosteroids
V3 (Mandibular branch of trigeminal)	3	(1) Local anesthetic effect causing sensory disturbances (2) Administration of corticosteroids
VII (Facial)—temporal and zygomatic branches	313	(1) Diffusion of local anesthetic resulting in transient motor blockadeor or (2) Extravasation of irrigation fluid causing compressive neurapraxia

## Data Availability

The protocol of this systematic review is available in the Prospective Register of Systematic Reviews (PROSPERO) under the number CRD420251088170. All collected data are included in the content of this article.

## Abbreviations

The following abbreviations are used in this manuscript:

TMDs	Temporomandibular disorders
TMJ	Temporomandibular joint

## Appendix A

**Table A1:** Number of records identified and database coverage on the day of the search.

Search Engine	Total Retrieval Range	Identified Records
Bielefeld Academic Search Engine (BASE)	423,038,726	53
Directory of Open Access Journals (DOAJ)	11,166,729	7
National Library of Medicine (PubMed)	Over 38,000,000	46
Scientific Electronic Library Online (SciELO)	14,388,173	1
Semantic Scholar	226,698,359	25

**Table A2:** Reports that were rejected at the full-text assessment stage.

First Author, Publication Year	Title	Digital Object Identifier (DOI) or Alternative	Reason for Exclusion
Huang, 2024 [39]	A clinical trial of ropivacaine in arthrocentesis for TMD	10.1186/s12903-024-04606-x	No neurological adverse events reported
Talaat, 2022 [40]	Minimally invasive surgeries for the treatment of temporomandibular disorders: Prognostic indicators and persistence of treatment outcomes over a 5-year follow-up	10.4103/abhs.abhs_14_21	Concomitant invasive co-intervention (arthroscopy)
Vervaeke, 2022 [41]	Correlation of MRI and arthroscopic findings with clinical outcome in temporomandibular joint disorders: a retrospective cohort study	10.1186/s13005-021-00305-y	No neurological adverse events reported
Ferreira, 2021 [42]	Clinical outcomes in TMD patients after arthrocentesis with lysis, lavage, and viscossupplementation	10.1080/07853890.2021.1897446	Conference proceeding; no neurological adverse events reported
Zhang, 2020 [43]	Gradient sequential treatment of temporomandibular joint disorders	10.12016/j.issn.2096-1456.2020.01.002	Review article
Patel, 2017 [44]	Clinical and radiological outcome of arthrocentesis followed by autologous blood injection for treatment of chronic recurrent temporomandibular joint dislocation	10.4317/jced.53812	No neurological adverse events reported
Bayoumi, 2014 [45]	Arthrocentesis followed by intra-articular autologous blood injection for the treatment of recurrent temporomandibular joint dislocation	10.1016/j.ijom.2014.05.004	No neurological adverse events reported
Etoz, 2010 [46]	Accidental use of alcohol during arthrocentesis of the temporomandibular joint	10.1016/j.bjoms.2010.11.010	Unintentional use of an inappropriate substance (alcohol)
Simpson, 1968 [47]	Auriculotemporal syndrome after injection into the temporomandibular joint: report of case	PMID: 4299382	Full text not retrieved

**Table A3:** Studies included in the review.

First Author, Publication Year	Title	Digital Object Identifier (DOI) or Alternative	Study Design
Baş, 2025 [27]	What Are the Complications of Temporomandibular Joint Arthrocentesis?	10.1016/j.joms.2024.12.012	Retrospective study
Carroll, 2000 [28]	Extradural haematoma following temporomandibular joint arthrocentesis and lavage	10.1080/02688690050004633	Case report
Aliyev, 2019 [29]	An unusual complication during arthrocentesis: *N. facialis* paralysis, with *N. lingualis* and *N. alveolaris* inferior anesthesia	10.17245/jdapm.2019.19.2.115	Case report
Isacsson, 2019 [30]	Pain relief following a single-dose intra-articular injection of methylprednisolone in the temporomandibular joint arthralgia—A multicentre randomised controlled trial	10.1111/joor.12718	Randomized controlled trial
Vaira, 2018 [31]	Complications and post-operative sequelae of temporomandibular joint arthrocentesis	10.1080/08869634.2017.1341138	Retrospective study

**Table A4:** Critical appraisal of the randomized controlled trial of Isacsson, 2019 [30].

JBI Critical Appraisal Criteria	Response
Was true randomization used for the assignment of participants to treatment groups?	Yes
Was allocation to treatment groups concealed?	Yes
Were treatment groups similar at the baseline?	Yes
Were participants blind to treatment assignment?	No
Were those delivering the treatment blind to treatment assignment?	Yes
Were treatment groups treated identically, other than the intervention of interest?	Yes
Were outcome assessors blind to treatment assignment?	Yes
Were outcomes measured in the same way for treatment groups?	Yes
Were outcomes measured in a reliable way?	Yes
Was the follow-up complete, and if not, were the differences between groups in terms of their follow-up adequately described and analyzed?	Yes
Were participants analyzed in the groups to which they were randomized?	Yes
Was an appropriate statistical analysis used?	Yes
Was the trial design appropriate, and were any deviations from the standard RCT design (individual randomization, parallel groups) accounted for in the conduct and analysis of the trial?	Yes

**Table A5:** Critical appraisal of analytical cross-sectional studies.

First Author, Publication Year	Were the Criteria for Inclusion in the Sample Clearly Defined?	Were the Study Subjects and the Setting Described in Detail?	Was the Exposure Measured in a Valid and Reliable Way?	Were Objective, Standard Criteria Used for Measurement of the Condition?	Were Confounding Factors Identified?	Were Strategies to Deal with Confounding Factors Stated?	Were the Outcomes Measured in a Valid and Reliable Way?	Was Appropriate Statistical Analysis Used?
Baş, 2025 [27]	Yes	Yes	Yes	Yes	Yes	Yes	Yes	Yes
Vaira, 2018 [31]	Yes	Yes	Yes	Yes	No	No	Yes	Unclear

**Table A6:** Critical appraisal of case reports.

First Author, Publication Year	Were the Patient’s Demographic Characteristics Clearly Described?	Was the Patient’s History Clearly Described and Presented as a Timeline?	Was the Current Clinical Condition of the Patient on Presentation Clearly Described?	Were Diagnostic Tests or Assessment Methods and the Results Clearly Described?	Was the Intervention(s) or Treatment Procedure(s) Clearly Described?	Was the Post-Intervention Clinical Condition Clearly Described?	Were Adverse Events (Harms) or Unanticipated Events Identified and Described?	Does the Case Report Provide Takeaway Lessons?
Carroll, 2000 [28]	Yes	Unclear	Yes	Yes	Yes	Yes	Yes	Yes
Aliyev, 2019 [29]	Yes	Yes	Yes	Yes	Yes	Yes	Yes	Yes
